# Butyrate Inhibits Colorectal Cancer Cell Proliferation through Autophagy Degradation of β-Catenin Regardless of *APC* and β-Catenin Mutational Status

**DOI:** 10.3390/biomedicines10051131

**Published:** 2022-05-13

**Authors:** Beatrice Garavaglia, Letizia Vallino, Alessandra Ferraresi, Andrea Esposito, Amreen Salwa, Chiara Vidoni, Sergio Gentilli, Ciro Isidoro

**Affiliations:** 1 Laboratory of Molecular Pathology, Department of Health Sciences, University of Piemonte Orientale “A. Avogadro”, Via Solaroli 17, 28100 Novara, Italy; beatrice.garavaglia@uniupo.it (B.G.); letizia.vallino@uniupo.it (L.V.); alesssandra.ferraresi@med.uniupo.it (A.F.); andrea.esposito@uniupo.it (A.E.); salwa.amreen@uniupo.it (A.S.); chiara.vidoni@med.uniupo.it (C.V.); 2 Department of Health Sciences, Division of General Surgery, Maggiore della Carità Hospital, University of Piemonte Orientale, 28100 Novara, Italy; sergio.gentilli@med.uniupo.it

**Keywords:** short chain fatty acid (SCFA), cell proliferation, WNT signaling pathway, microbiota, autophagy, LC3, gene correlation analysis, probiotics, inflammation

## Abstract

Colorectal cancer (CRC) pathogenesis is mainly driven by alterations in WNT signaling, which results in altered transcriptional activity of β-Catenin. Mutations in *APC* (Adenomatous Polyposis Coli) are reflected in β-Catenin hyperactivation and loss of proliferation control. Certain intestinal bacteria metabolites have shown the ability to limit CRC cell proliferation and CRC pathogenesis. Here, we investigated the molecular mechanism underlying the anti-proliferative activity of butyrate, a microbiota-derived short chain fatty acid, in two CRC cell lines, namely HCT116 and SW620, which bear a mutation in β-Catenin and *APC*, respectively. In particular, we focused on autophagy, a lysosome-dependent degradation pathway, which was shown to control intestinal tissue homeostasis. Butyrate reduced CRC cell proliferation, as witnessed by the downregulation of proliferation markers. TCGA bioinformatic transcriptomic analysis of *CTNNB1* (β-Catenin) gene correlation in CRC patients showed that β-Catenin negatively correlates with the autophagy gene *ATG4D*. In CRC cells, regardless of the mutational state of *APC* or β-Catenin genes, butyrate caused the autophagy-mediated degradation of β-Catenin; thus, preventing its transcriptional activity. Autophagy gene silencing restored β-Catenin levels, allowing it to translocate into the nucleus to promote the expression of downstream genes associated with cancer cell proliferation. CRC-affected patients show driver mutations in the WNT pathway; thus, targeting its crucial effector may be a promising therapeutic strategy in CRC treatment; for instance, by using *ad hoc* probiotics that stimulate autophagy.

## 1. Introduction

Colorectal cancer (CRC) is the third most common cancer and the fourth most common cause of cancer-related death globally, with one million new cases diagnosed every year and 600,000 deaths per year, the incidence being higher in developed countries [1].

CRC is a heterogeneous disease at genetic level, due to the many different mutations occurring in the epithelium of the large intestine [2], and an early event in the adenoma to adenocarcinoma transformation is the mutation in the tumor suppressor gene Adenomatous Polyposis Coli (*APC*) [3].

Interestingly, the canonical WNT/β-Catenin pathway has been found to be constitutively activated in 80% of colorectal tumors [4], and the primary genetic alteration causing such activation is the loss of function of *APC* [5]. Consistently, *APC* mutation has been linked to intestinal microbiota dysbiosis [6]. The stability of β-Catenin is regulated by the assembly of the destruction complex. In the absence of the Wnt ligand, the cytoplasmic β-Catenin protein is sequestered by the complex formed by the tumor suppressor APC, axin inhibitor (Axin), casein kinase 1 (CK1), and glycogen synthase kinase-3β (GSK3β) [7]. When the pathway is triggered, β-Catenin phosphorylation and proteasomal degradation are prevented [8]. This leads to increased levels of cytoplasmic β-Catenin and its translocation into the nucleus, where it promotes the transcription of target genes [9,10]. Mutations in the phosphorylation sites of β-Catenin lead to its cellular accumulation and associate with cell malignancy [11]. A similar outcome arises from mutations in the APC domain for interacting with β-Catenin [12]. These mutations seem to not influence cell proliferation [13]. However, it remains to be determined how these mutations influence the response of CRC cells to microbiota-derived metabolites that have shown anti-CRC activity. In addition, the fate of β-Catenin mutants lacking the domain for phosphorylation, driving proteasome degradation in CRC cells exposed to such microbiota metabolites, is not known.

Here, we addressed these issues, employing two CRC cell lines, namely HCT116 and SW620, that express a wild-type *APC,* along with β-Catenin mutated in the phosphorylation domain, and a truncated APC mutant, along with wild-type β-Catenin, respectively [13,14,15,16,17].

We investigated the fate of β-Catenin in these cells exposed to butyrate, the microbiota metabolite that has been shown to prevent CRC through inhibition of the Wnt signaling [18]. We hypothesized the involvement of the autophagy-lysosomal pathway as a possible proteolytic pathway, alternative to proteasome for β-Catenin degradation in CRC cells exposed to butyrate.

Autophagy (herein meaning macroautophagy) is a catabolic lysosome-driven process that is crucial for the maintenance of cellular homeostasis and found to be deregulated in cancer [19]. Autophagy deregulation may lead to gut microbiota dysbiosis and generate a chronic immune cell activation, which results in exacerbated intestinal inflammation, thus increasing the risk of colon cancer [20]. We and others have shown that autophagy plays a role in the control of CRC growth and apoptosis [21,22,23]. Of relevance, there is a complex cross-talk between the canonical Wnt pathway and autophagy in cancer cells [24], where β-Catenin suppresses autophagy, while it is degraded by starvation-induced autophagy [25].

Here we show that butyrate counteracts CRC cell proliferation through induction of autophagy. Accordingly, interrogation of the cancer genome atlas (TCGA) transcriptome in CRC patients revealed that the β-Catenin (*CTNNB1*) gene negatively correlated with the autophagy gene *ATG4D*. Notably, butyrate promotes the interaction of β-Catenin with LC3, indicating its sequestration within the autophagosome. The genetic silencing of the autophagy gene *ATG7* restores the level of β-Catenin and abrogates the cell cycle arrest in CRC cells exposed to butyrate, confirming that, in CRC cells bearing mutations in the machinery for the degradation of β-Catenin, this probiotic metabolite still elicits anti-proliferative activity, by activating an alternative degradation of the downstream effector of the WNT pathway. The present findings may translate to clinics through the designing of probiotic supplements that enhance autophagy in intestinal cancer cells as pre- and post-operative adjuvant therapies.

## 2. Materials and Methods

### 2.1. Cell Culture and Treatments

Human colorectal cancer HCT116 (CCL-247™) and SW620 (CCL-227™) cell lines, with different genetic background (Table 1) and tumor site origins, were purchased from the American Type Culture Collection (ATCC). HCT116 and SW620 cell lines were maintained in standard culture conditions (37 °C, 95 *v/v*% air: 5 *v/v*% CO_2_) in Dulbecco’s Modified Eagle Medium (DMEM, cod. D5671; Sigma-Aldrich, St. Louis, MO, USA) and RPMI-1640 Medium (RPMI, cod. R8758; Sigma-Aldrich, St. Louis, MO, USA), respectively, supplemented with 10% heated-inactivated fetal bovine serum (FBS, cod. ECS0180L; Euroclone, Milano, Italy), 1% glutamine (cod. G7513; Sigma Aldrich, St. Louis, MO, USA) and 1% penicillin/streptomycin (cod. P0781; Sigma-Aldrich, St. Louis, MO, USA). Treatments included 50 ng/mL interleukin-6 (IL-6, cod. 11340066; Immunotools, Friesoythe, Germany), 1 mM and 2 mM sodium butyrate (NaB, cod. B5887; Sigma-Aldrich, St. Louis, MO, USA), and 30 μM chloroquine (ClQ, cod. C-6628; Sigma-Aldrich, St. Louis, MO, USA). All three reagents were dissolved in sterile water.

### 2.2. Cell Counting and Cell Cycle Analysis

Cells were plated in p35 petri dishes at the density of 40,000–50,000 cells/cm^2^. The day after, cells were counted (time 0) and then treated with 2 mM sodium butyrate for 24, 48, and 72 h. At the end of each experimental time point, cells were trypsinised and collected, and cell suspension was diluted 1:1 with trypan blue (cod. T8154; Sigma-Aldrich, St. Louis, MO, USA), a dye that stains dead cells. Cell counting was performed in triplicate for each experimental condition. For cell cycle analysis, cells were fixed in 70% ice-cold ethanol and stored at −20 °C. Before starting the cytofluorimetric analysis, RNAse (50 μg/mL) was added to cells for 30 min at 37 °C. Cells were stained with propidium iodide (PI, 50 μg/mL; cod. P4170, Sigma-Aldrich, St. Louis, MO, USA) and acquired using a FacScan flow cytometer (FACSCalibur, Becton Dickinson, Eysins, Switzerland). A fraction of 5000 events was analyzed for each sample. The processing of the data obtained was performed using the free tool Flowing Software 2.5.1 (Turku Center for Biotechnology, University of Turku, Finland).

### 2.3. Antibodies

The following primary antibodies (at the dilution indicated) were used for either immunofluorescence or Western blotting: rabbit anti-Ki67 (1:100, cod. HPA001164; Sigma-Aldrich, St. Louis, MO, USA); mouse anti-p21 (1:100, cod. B1313; Santa Cruz Biotechnology, Dallas, TX, USA); mouse anti-Histone H3 (1:500, cod. 61475; Active motif, Carlsbad, San Diego, CA, USA); mouse anti-LC3 (1:100, cod. NB600-1384; Novus Biologicals, Milano, Italy); rabbit anti-LC3 (1:1000, cod. L7543; Sigma-Aldrich, St. Louis, MO, USA); mouse anti-LAMP1 (1:1000, cod. 555798; BD, Biosciences, Franklin Lakes, NJ, USA); rabbit anti-β-Catenin (1:500, cod. PA5-77934; Invitrogen, Paisley, UK); rabbit-anti ATG7 (1:500, cod. AB10511; Millipore, Burlington, MA, USA); mouse-anti ATG7 (1:500, cod. SAB4200304; Sigma-Aldrich, St. Louis, MO, USA); mouse anti-β-tubulin (1:1000, cod. T5326; Sigma-Aldrich, St. Louis, MO, USA); rabbit anti-GAPDH (1:1000, cod. G9545; Sigma-Aldrich, St. Louis, MO, USA); mouse anti-β-actin (1:2000, cod. A5441; Sigma-Aldrich, St. Louis, MO, USA).

### 2.4. Western Blotting Analysis

Cells were seeded in p35 petri dishes at the density of 40,000–50,000 cells/cm^2^ and treated as indicated when confluence reached approximately 80%. Cell homogenates were prepared by freeze–thawing and ultrasonication in Ripa lysis buffer containing protease inhibitors. Equal amounts of proteins (25 μg) from total lysates were separated on SDS-PAGE and then transferred onto PVDF membranes (cod. 03010040001; Sigma-Aldrich, St. Louis, MO, USA). The membranes were blocked with 5% non-fat dry milk (cod. A0830; PanReac, Castellar den Vallès, Barcellona, Spain) +0.2% Tween for 1 h at room temperature. Then, membranes were incubated with specific primary antibodies overnight at 4 °C, followed by incubation with secondary HRP-conjugated antibodies (goat anti-mouse cod. 1706516; goat anti-rabbit cod. 1706515, BioRad, Hercules, CA, USA) for 1 h at room temperature. The bands were detected using enhanced Chemiluminescence reagents (ECL, cod. NEL105001EA; Perkin Elmer, Waltham, MA, USA) and developed with a ChemiDoc XRS instrument (BioRad, Hercules, CA, USA). Intensity of the bands was estimated by densitometry using Image Lab 6.0 (BioRad, Hercules, CA, USA). Western blotting data were reproduced at least three times, separately.

### 2.5. Immunofluorescence Assay

Cells were seeded onto sterile coverslips at the density of 25,000–30,000 cells/cm^2^, left to adhere, and treated as reported. At the end of the experiment, the coverslips were fixed within ice-cold methanol, permeabilized with 0.2% Triton-PBS, and incubated overnight at 4 °C with specific primary antibodies dissolved in 0.1% Triton-PBS + 10% FBS. The following day, coverslips were incubated for 1 h at room temperature with secondary antibodies (diluted in 0.1% Triton-PBS + 10% FBS), and either AlexaFluor488-conjugated goat-anti rabbit IgG (1:000, cod. A32731, Invitrogen, Paisley, UK) or AlexaFluor555-conjugated goat-anti mouse IgG (1:1000, cod. A32727, Invitrogen, Paisley, UK), as appropriate. Nuclei were stained with UV fluorescent dye DAPI (4′,6-diamidino-2-phenylindole). Coverslips were mounted onto glass using SlowFade reagent (cod. S36936; Life Technologies, Paisley, UK) and imaged with a fluorescence microscope (Leica Microsystems DMI6000; Wetzlar, Germany).

### 2.6. 3D-Spheroid Forming Assay

Poly(2-hydroxyethylmethacrylate) or polyhema (cod. P3932; Sigma-Aldrich, St. Louis, MO, USA) was dissolved in 95% ethanol and left under rotation overnight at 50 °C. The stock solution was diluted in ethanol to obtain a working solution of 120 mg/mL. P35 petri dishes were coated with this solution, left under a biological hood to completely dry, and then stored at room temperature until used. Cells were seeded at the density of 500,000 cells/p35 and treated according to the experimental conditions. The growth of spheroids was monitored by taking pictures with a phase-contrast microscope at each time point. The quantification of the dimensions of the spheroids was analyzed with ImageJ software 1.51 (National Institutes of Health, MD, USA).

Three-dimensional immunofluorescence was performed on spheroids cytospotted on glass slides at day 5. Spheroids were fixed in methanol, permeabilized for 30 min in 0.5% Triton-PBS, and processed as described above [26].

### 2.7. DiD Proliferation Assay

To assess cell proliferation in 3D spheroids, we employed plasma membrane staining with a vital fluorescent dye that undergoes dilution with cell division. Cell lines were stained with 1μM DiD (cod. V22887; Life Technologies, Paisley, UK) in serum-free medium at 37 °C for 30 min, then dissolved in complete medium, plated as described above for 3D spheroids, and treated as indicated. At day 0 and day 5 the spheroids were cytospotted on glass slides. The intensity of Did retention was immediately acquired with a fluorescence microscope (Leica Microsystems, DMI6000; Wetzlar, Germany) and then quantified with the software ImageJ 1.51 (National Institutes of Health, MD, USA).

### 2.8. Co-Immunoprecipitation Assay

This assay has previously been described in detail [27]. Essentially, adherent cells treated as indicated were pre-treated 20 min before collection with 1 mM of the chemical cross-linker 3-3′-dithiodipropionic acid di-(N-hydroxysuccinimide ester) (DTSP, cod. D3669, Sigma Aldrich, St. Louis, MO, USA). The same amount of protein (250 μg) was incubated with anti-LC3 antibody (2.5 μg) for at least 1 h at 4 °C, under rotation. To capture the immunocomplexes, 50 μL of Sepharose G beads (cod. P3296; Sigma-Aldrich, St. Louis, MO, USA) was added to each sample and left under rotation overnight at 4 °C. Immunocomplexes were then precipitated by centrifugation (1000 g) and eluted with 40 μL of Leammli buffer 1× at 95 °C for 10 min. An equal volume of eluate was loaded on SDS-PAGE and immunoblotted with specific antibodies to reveal the presence LC3 interactors.

### 2.9. Small Interference RNA Transfection

Post-transcriptional silencing of *ATG7* was performed with small interference RNA (siRNA) technology in cells cultivated on coverslips (for immunofluorescence purpose) or in petri dishes (for Western blotting purpose). Cells were plated on coverslips at the density of 20,000–30,000 cells/cm^2^ and on petri dishes at the density of 50,000–60,000 cells/cm^2^ and left to adhere for 24 h before transfection. Cells were transfected with siATG7 (siRNA sequences: scramble siRNA 5′-AGG UAG UGU AAU CGC CUU GTT-3′; *ATG7* siRNA 5′-GGG UUA UUA CUA CAA UGG UGT T-3′) using Lipofectamine 3000 Reagent (cod. 100022052, Invitrogen), as described previously [26]. The siRNA-loaded liposomal complexes were prepared in Opti-MEM I reduced serum medium (cod. 11058-021; Life Technologies, Paisley, UK) with 150 pmol siRNA and 7.5 µL of Lipofectamine 3000 (cod. L3000-015; Invitrogen, Paisley, UK) for each petri dish. After 6 h, the medium was replaced with complete serum-containing culture medium. Treatments were performed 36 h after the transfection and followed up to a maximum of 48 h. Culture medium was renewed every 24 h, to prevent secondary effects due to starvation. Coverslips and cell homogenates were then processed for immunofluorescence and Western blotting, respectively.

### 2.10. Bioinformatic Analysis

RNA-seq and clinical data of 526 colorectal adenocarcinoma (TCGA, PanCancer Atlas) patients were downloaded from the TCGA data repository (https://cancergenome.nih.gov, accessed on 2 October 2021). The mRNA gene expressions were downloaded from cBioportal.org [28].

RNA-seq data of colorectal adenocarcinoma samples were downloaded from the TCGA database (http://cancergenome.nih.gov/, accessed on 5 October 2021). In the current study, *CTNNB1* gene expression was correlated with 20,040 other groups of genes using Spearman’s correlation coefficient analysis and *p*-values were obtained. TBtools (https://github.com/CJ-Chen/TBtools/, accessed on 5 October 2021) was used to identify the differentially expressed genes (DEGs) in correlation with *CTNNB1,* which are represented in the form of Volcano plots. To identify the DEGs, cut-off criteria were set based on Spearman’s correlation value (i.e., correlation coefficient value greater than +0.25 (positively correlated) or lower than −0.25 (negatively correlated) and *p*-value < 0.0001 (−Log10 (*p*-value) threshold was fixed above 5.0) [29]. The DAVID bioinformatics functional annotation tool (https://david.ncifcrf.gov/summary.jsp, accessed on 10 October 2021) was used to analyze gene ontology (GO) biological processes, and Kyoto Encyclopedia of Genes and Genomes (KEGG) pathways were obtained with the help of positive and negative-differentially expressed genes. Data are presented in bar graphs showing the number of transcripts belonging to each positively and negatively associated biological process [30].

### 2.11. Statistical Analysis

All data refer to at least three separate experiments performed by different operators. Data in histograms are shown as average ± S.D. GraphPad Prism was employed (GraphPad Software Inc.) for statistical analysis. The statistical significance of the data was given by t student test and one-way ANOVA analysis of variance followed by Bonferroni’s test. Differences were considered statistically significant for * = *p* < 0.05; ** = *p* < 0.01; *** = *p* < 0.001; **** = *p* < 0.0001.

## 3. Results

### 3.1. Butyrate Induces CRC Cell Growth Arrest

We searched the TCGA database for the presence of altered *APC*, β-Catenin (*CTNNB1*) and *GSK3β* in 526 colorectal adenocarcinoma patient samples (TCGA, PanCancer Atlas). The oncoprint (Figure S1) shows that *APC* presents an alteration in 75% of the cases, β-Catenin (*CTNNB1*) is altered in 7% of the cases, and *GSK3β* is altered in just 2.5% of cases. Moreover, the comparison of available CRC cell lines shows the presence of *APC* or β-Catenin (*CTNNB1*) mutations, confirming that these mutations are mutually exclusive. Additionally, genetic mutations of *GSK3β* are absent or undetectable [31]. Therefore, in this study we chose as a CRC cell model the cell lines HCT116 and SW620 bearing *APC* or β-Catenin (*CTNNB1*) genetic alterations, respectively, as representative of the majority of CRC diagnosed.

**Figure 1:**
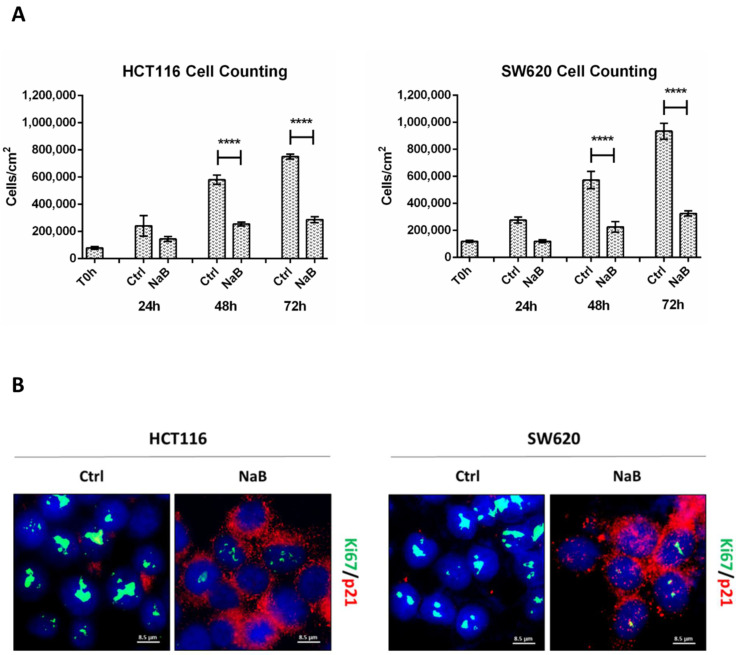

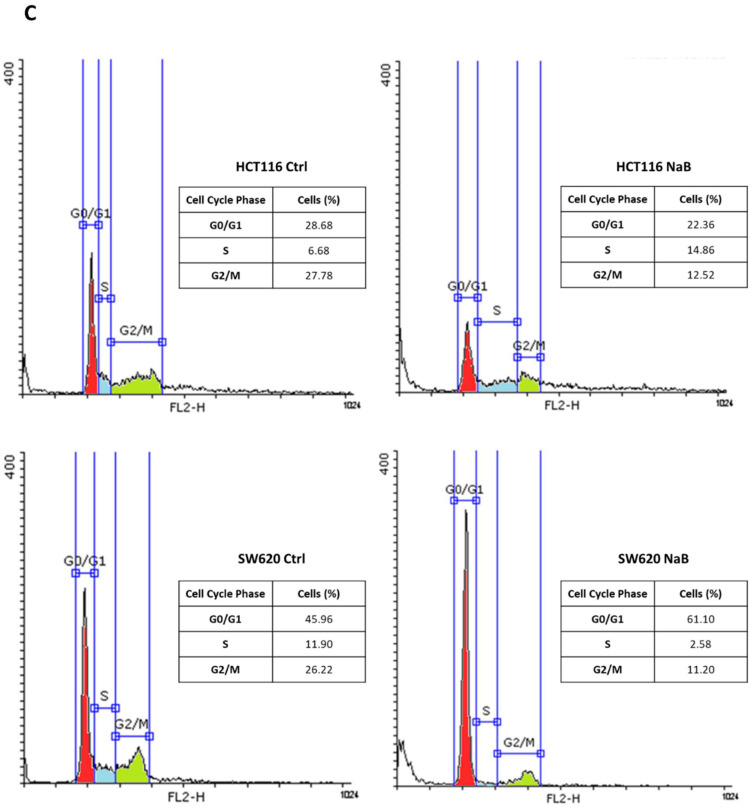
Sodium butyrate (NaB) inhibits colorectal cancer (CRC) cell proliferation. (**A**) HCT116 and SW620 cells were incubated with 2 mM NaB for the indicated time points. Cell counting was performed up to 72-h of treatment. At the end of each experimental time point, the cell suspension was diluted 1:1 with trypan blue and counted. The graph represents the average ± SD calculated in triplicate for each sample (significance was considered as follow: **** *p* < 0.0001). (**B**) HCT116 and SW620 cells adhering to the coverslip were treated with 2 mM NaB for 48 h. Medium was replaced and NaB re-added at 24 h. At the end of the treatment, coverslips were processed for immunofluorescence staining of Ki67 (green) and p21 (red). Nuclei were stained with DAPI. The cells were photographed under fluorescence microscopy (scale bar = 8.5 μm, magnification = 63×). (**C**) HCT116 and SW620 cells were incubated with 2 mM NaB for the indicated time. Cytofluorimetric analysis of the cell cycle was performed at 48 h using a FacScan flow cytometer. The area under each peak shows a different fraction of cell population in the respective cell cycle phase. Cell cycle analysis was performed in triplicate for each experimental condition. A representative analysis out of three for each condition, with reproducible data, is shown.

The cell counting of viable (trypan blue-excluding) HCT116 and SW620 colorectal cancer (CRC) cells exposed to 2 mM sodium butyrate (NaB) showed a large decrease in cell population, compared to untreated culture counterparts, from 48 to 72 h (Figure 1A). We performed immunofluorescence to monitor the expression of (i) Ki67, a nuclear antigen expressed only in actively proliferating cells, and (ii) p21waf/Cip1, a potent cyclin-dependent kinase inhibitor and key regulator of the G1/S checkpoint. HCT116 and SW620 treated with NaB presented an increased accumulation of p21 in parallel with reduced Ki67 expression, suggesting the arrest in the G1/S transition phase of the cell cycle, while untreated cells displayed an increased level of Ki67 suggestive of cell proliferation (Figure 1B). Cytofluorimetric analysis (Figure 1C) revealed that NaB increases the proportion of cells in S phase in HCT116 (from 6.68% to 14.86%) by preventing their transition in the mitotic phase, and it increases the proportion of cells in G0/G1 phase in SW620 (from 45.96% to 61.10%) by preventing the entering in the S phase. Thus, when exposed to butyrate, both HCT116 and SW620 cells cannot complete the cell cycle, as indicated by the fact that the proportion of cells in the G2/M decreases. We could also rule out that NaB was inducing cell senescence, since senescence-associated β-galactosidase (SA-βgal) activity was not increased (Figure S2).

### 3.2. Butyrate Counteracts IL-6-Induced 3D Colorectal Cancer Spheroid Growth

We moved to a 3D spheroid model that better mimics the in vivo tumor growth [32]. Furthermore, to partially mimic the inflammatory tumor microenvironment, we introduced interleukin-6 (IL-6), which is the cytokine most reported to stimulate the growth of CRC cells [33,34].

**Figure 2:**
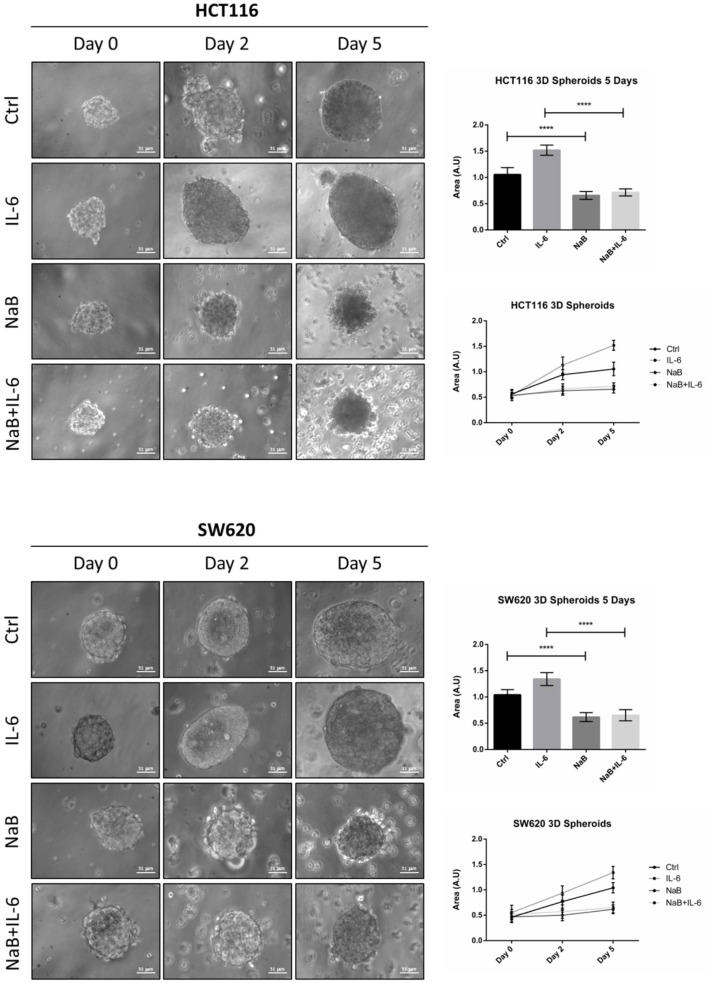
Sodium butyrate reduces the growth of 3D CRC spheroids. HCT116 and SW620 cells growing in suspension in polyhema-coated petri dishes were treated with 1 mM NaB and 50 ng/mL interleukine-6 (IL-6), alone or in combination. Medium and substances were replaced every 48 h. The growth of spheroids was monitored by taking pictures with a phase contrast microscope up to 5 days (scale bar = 31 μm). Images representative of each condition are shown. The graphs represent the average area ± SD (significance was considered as follow: **** *p* < 0.0001).

We monitored the ability of colon cancer cells to form spheroids for up to 5 days. Butyrate impaired the growth of CRC spheroids, as indicated by the fact that the diameter remained the same at day 0. IL-6 increased the dimension of CRC spheroids starting from day 2, and this effect was significantly counteracted by NaB (Figure 2 shows representative images of spheroid shape and dimension; average area of the spheroids is also shown). The effect of butyrate could result from a cell proliferation impairment or a balanced cell proliferation/cell death rate.

**Figure 3:**
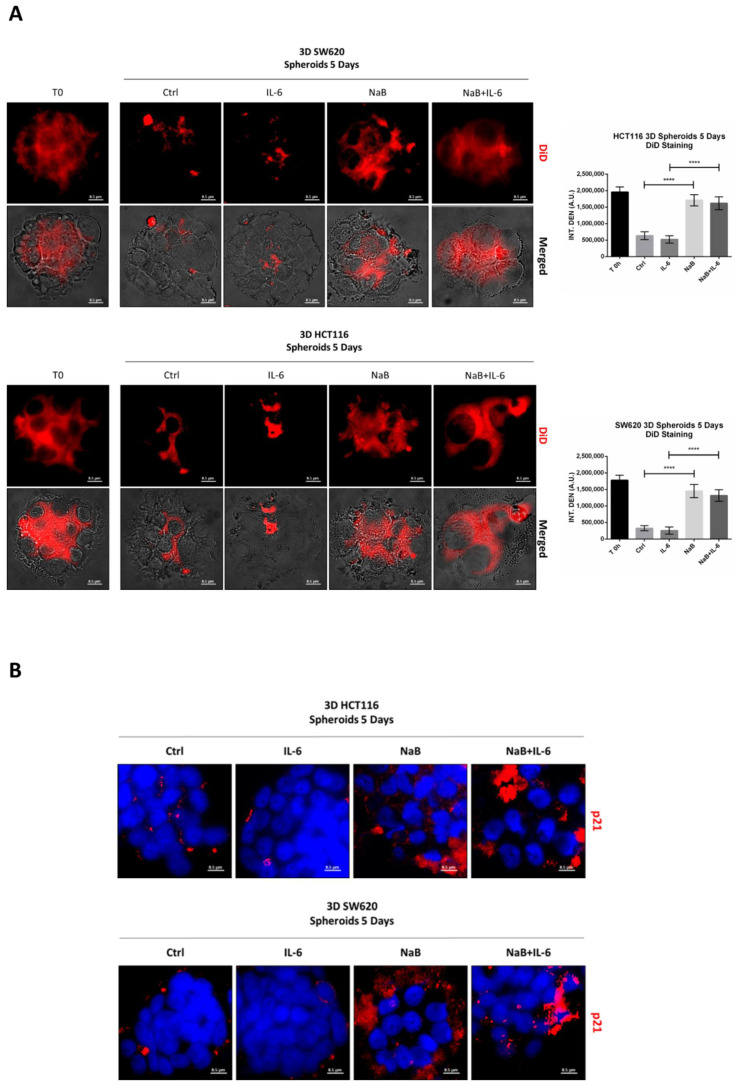

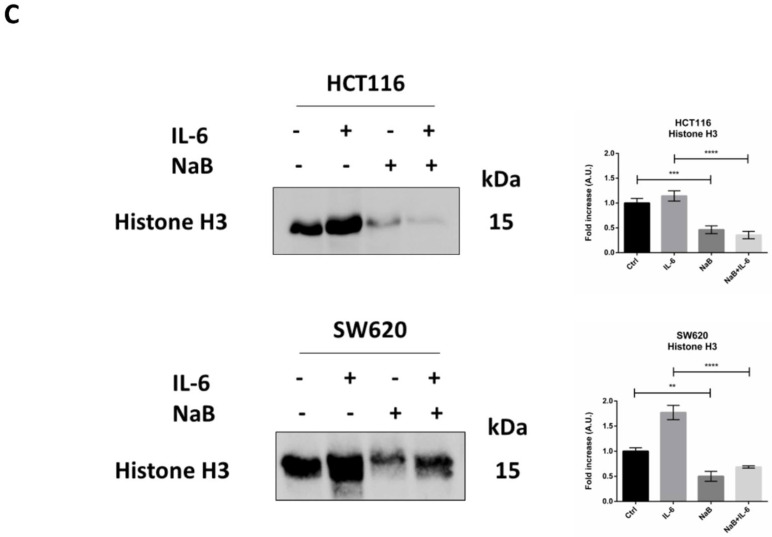
Sodium butyrate opposes IL-6-induced CRC cell proliferation. (**A**) CRC cells growing in polyhema-coated petri dishes were stained with 1 μM DiD and treated with 1 mM NaB in the presence or not of 50 ng/mL IL-6. The intensity of Did retention in spheroids was acquired with a fluorescence microscope (scale bar = 8.5 μm, magnification = 63×) at days 0 and 5 and quantified with the software ImageJ. A representative microscopic field for each condition is shown. The graph represents the average fluorescence ± SD (significance was considered as follow: **** *p* < 0.0001). (**B**) Cell lines were cultured and treated as above. After 5 day-treatment, spheroids were stained for the proliferation marker p21 (red) and acquired at the fluorescence microscope (scale bar = 8.5 μm, magnification = 63×). Representative images for each condition are shown. (**C**) Spheroid homogenates were processed using Western blotting. An equal volume of homogenate (35 μL) was loaded to evaluate the expression of the nuclear marker histone H3 after 5 days of incubation with NaB and/or IL-6. A representative Western blotting out of three, with reproducible data, is shown and densitometric data are shown in the panel. The graphs represent the average ± SD (significance was considered as follow: ** *p* < 0.01, *** *p* < 0.001, **** *p* < 0.0001).

To confirm the anti-proliferative effect of NaB on CRC spheroids, the cells were preloaded with the fluorescent dye DiD, whose retention into phospholipid cell membrane and subsequent dilution upon cell division allows monitoring in vitro the proliferation rate of a cell population. Figure 3A shows that the DiD fluorescence in HCT116 and SW620 cells decreased by day 5, and it further decreased in the spheroid cultures incubated with IL-6, indicative of intense cell proliferation. By contrast, in the spheroid cultures incubated in presence of NaB, the cells showed a high retention of the DiD fluorescent signal, indicating the slowing down of cell division, and this also occurs when co-treated with IL-6. This interpretation was supported by the immunofluorescence staining of p21, whose expression was strongly increased in NaB-treated, while it was decreased in IL-6-treated, cells (Figure 3B). As an alternative to cell counting, which is objectively difficult to perform and not reliable when dealing with 3D spheroid suspensions, we determined by Western blotting the expression of the nuclear protein Histone H3, which reflects cell numbers. Consistent with the data on spheroid dimension (Figure 2), the content of this marker increased in 3D spheroid cultures incubated with IL-6, while it was reduced to levels half that of the controls when NaB was added to the culture, this effect being evident even in IL-6 co-treated cultures (Figure 3C).

### 3.3. Identification of Differentially Expressed Genes in Correlation with CTNNB1

**Figure 4:**
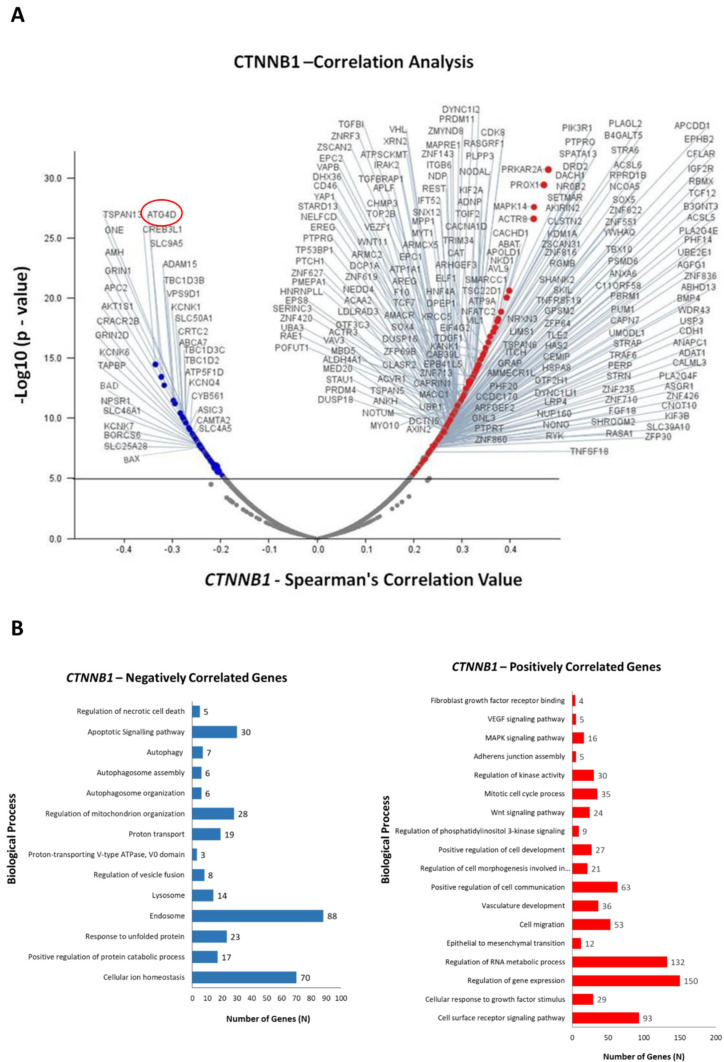
Differential modulation of biological processes by *CTNNB1* (β-Catenin) in colorectal adenocarcinoma patients. (**A**) Volcano plot displaying the differentially expressed genes (DEGs). Red dots represent *CTNNB1*-positively correlated genes (logFC/correlation value: +0.25), while blue dots represent *CTNNB1*-negatively correlated genes (logFC/correlation value: −0.25). (**B**) The bar graphs report the biological processes negatively (blue) and positively (red) correlated with *CTNNB1*.

To obtain an insight into the functional role of *CTNNB1* (β-Catenin) gene expression in CRC growth, we performed an in-silico transcriptomic analysis in a dataset of colorectal adenocarcinoma patients. We retrieved the RNA-Seq data (mRNA expression profile) from the TCGA database (PanCancer Atlas dataset) and performed a co-expression analysis to identify the genes that are positively and negatively correlated with *CTNNB1*. Up to 1000 differentially expressed genes (DEGs) were identified, out of which 501 genes were positively correlated (up-regulated) and 499 genes negatively correlated (down-regulated) with *CTNNB1*. We selected the most significant 250 DEGs, which are represented in the Volcano plot reported in Figure 4A. Next, we investigated more in depth the biological processes associated with the *CTNNB1*-positively and *CTNNB1*- negatively regulated DEGs. As shown in Figure 4B, *CTNNB1*-positively correlated genes are significantly enriched for the regulation of the mitotic cell cycle, cell proliferation, angiogenesis, cell-to-cell communications, response to growth factor signals, cell migration, and epithelial to mesenchymal transition. In more detail, these genes are associated to PI3K (phosphatidylinositol 3-kinase), MAPK (mitogen-activated protein kinase), and Wnt signaling pathways. In contrast, the *CTNNB1*-negatively correlated genes are significantly enriched for the regulation of apoptotic, proteolytic, and autophagy-lysosomal pathways. Of relevance to our initial hypothesis, high expression of *CTNNB1* correlated with low expression of *ATG4D* gene, which codes for the autophagy protein ATG4 that contributes to autophagosome building by processing MAP-LC3 [35].

### 3.4. Butyrate Induces the Autophagosomal Sequestration and Subsequent Degradation of β-Catenin by Promoting Its Interaction with LC3

**Figure 5:**
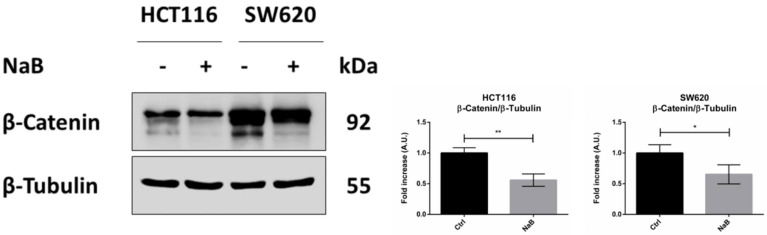
Sodium butyrate decreases β-Catenin cellular content. HCT116 and SW620 cells were incubated with 2 mM NaB for 48 h. Medium was replaced and substances re-added at 24 h to avoid possible starvation effects. Cell homogenates were analyzed by Western blotting for the expression of β-Catenin. The filter was re-probed with β-Tubulin as a loading control. The blot is representative of three experiments with reproducible data; band densitometry is shown in the panel. The graphs represent the average ± SD (significance was considered as follow: * *p* < 0.05, ** *p* < 0.01).

In line with our hypothesis and based on the gene correlation analysis, we then investigated whether butyrate exerted its anti-proliferative action on CRC cells by inducing β-Catenin degradation and whether this was carried out via autophagy. As shown in Figure 5, the level of β-Catenin was nearly halved in the cells incubated for 48 h with butyrate.

**Figure 6:**
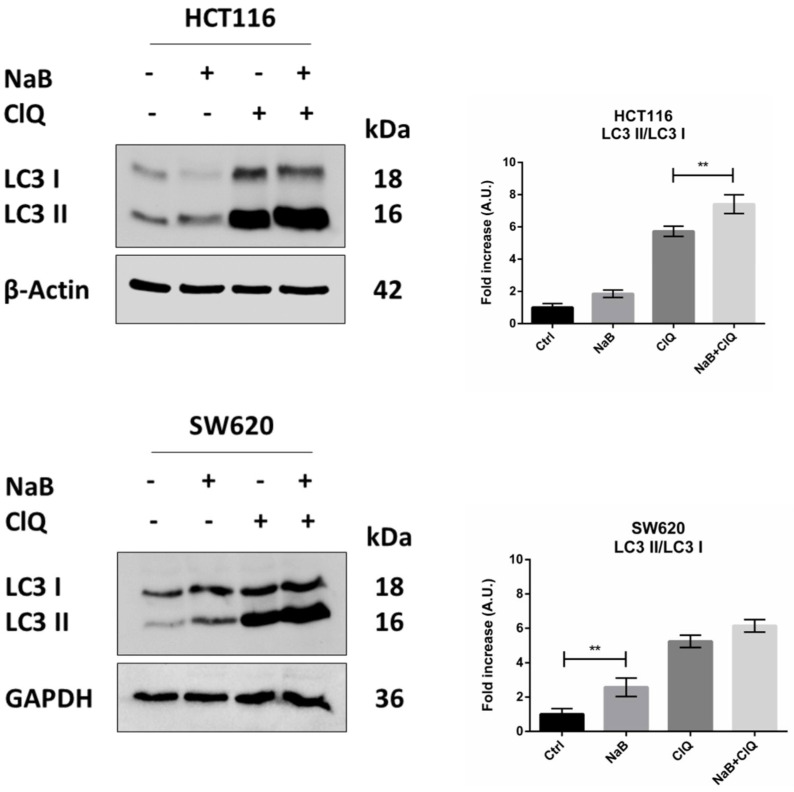
NaB stimulates the processing of LC3. HCT116 and SW620 cells adherent on petri dishes were incubated with 2 mM NaB for 48 h in the presence or in the absence of 30 μM chloroquine (added 24 h before cell collection). Medium was replaced and substances re-added every 24 h. At the end of the experimental time point, cell homogenates were analyzed by Western blotting for the expression of LC3. The filter was re-probed with β-Actin or GAPDH as a loading control. A representative Western blot out of three, with reproducible data, is shown, and densitometric data are shown in the panel. The graphs represent the average ± SD (significance was considered as follow: ** *p* < 0.01).

Next, we investigated whether and how autophagy is modulated by butyrate in CRC cells. The treatment with butyrate induced the conversion of the cytosolic LC3-I isoform into the autophagosome-associated LC3-II isoform, suggestive of autophagy (Figure 6). An increase in the LC3-II/LC3-I ratio was also observed in the presence of chloroquine, which blocks the fusion between autophagosome and lysosome, indicating that butyrate induced the neo-genesis of autophagosomes. Accordingly, the absolute content of LC3-I and LC3-II increased several fold in the cells treated with butyrate, and this increase was little, if at all, further increased in the presence of chloroquine, indicating that butyrate induced neo-synthesis of LC3 (Figure 6).

**Figure 7:**
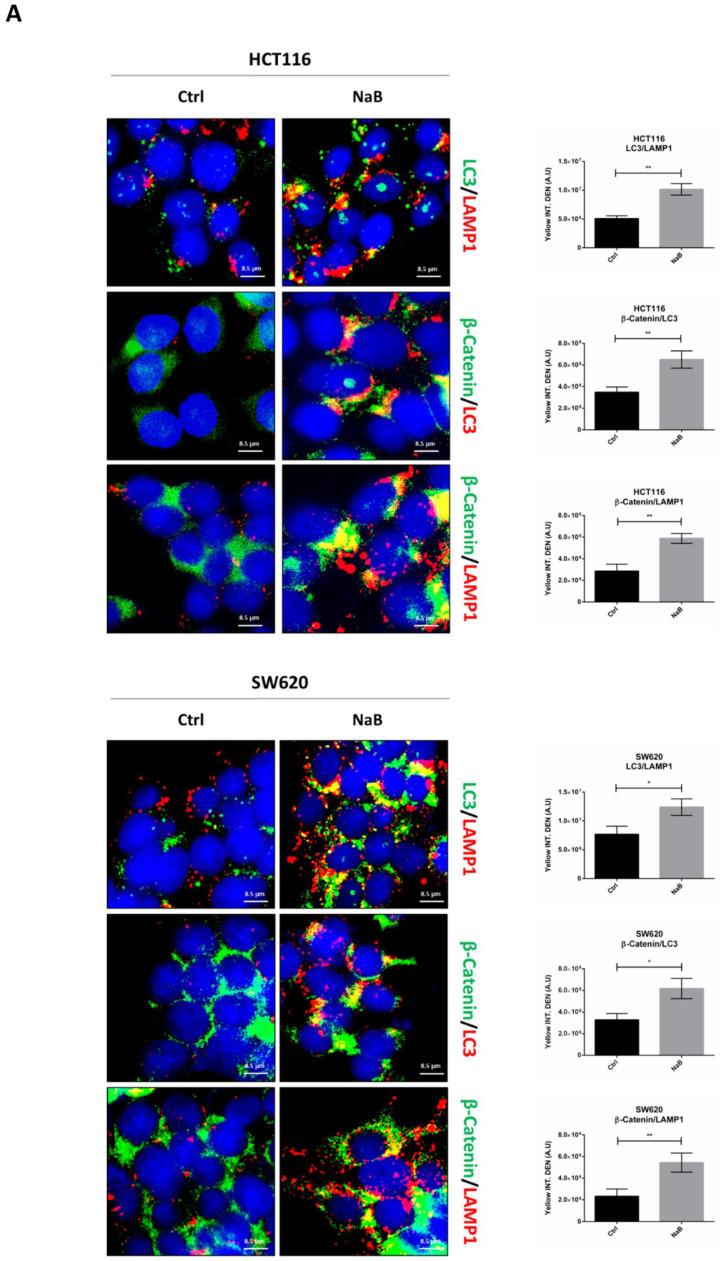

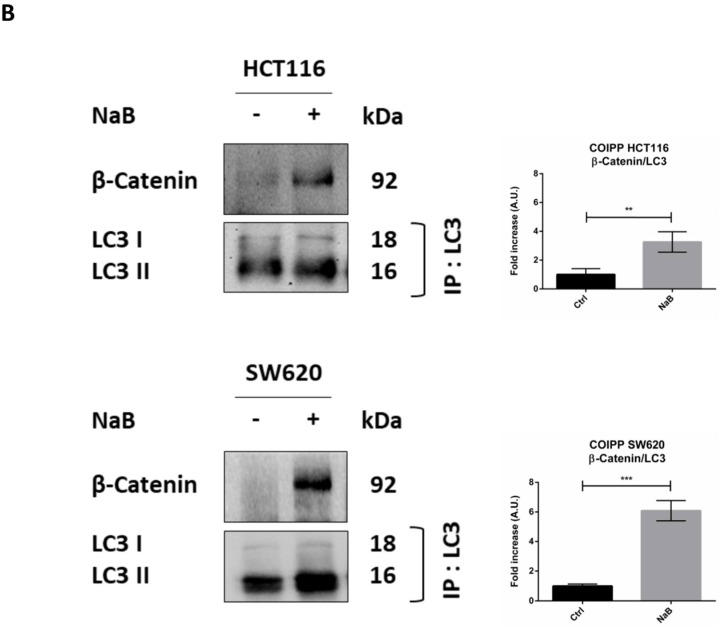
Sodium butyrate induces β-Catenin sequestration in the autophagy-lysosomal system. (**A**) HCT116 and SW620 cells adhering to coverslips were treated as indicated for 48 h, thereafter cells were fixed and double-stained for LC3 (green)/LAMP1 (red), β-Catenin (green)/LC3 (red), or for β-Catenin (green)/LAMP1 (red). Nuclei were stained with DAPI. The images were acquired by fluorescence microscope (scale bar = 8.5 μm, magnification = 63×). The images shown are representative of various fields for each condition. Bars in the graph indicate the average yellow fluorescence integrity density. Data are from three different images for each condition. The graphs represent the yellow integrity density ± SD (significance was considered as follow: * *p* < 0.05, ** *p* < 0.01). (**B**) A parallel set of cultures in petri dishes was processed for co-immunoprecipitation analysis of LC3 interacting protein. LC3 and β-Catenin were revealed by Western blotting. The experiment was performed three times with similar results. Densitometric data are shown in the panel. The graphs represent the average ± SD (significance was considered as follow: ** *p* < 0.01, *** *p* < 0.001).

We then investigated whether autophagy was responsible for the reduced level of cellular β-Catenin in butyrate-treated CRC cells. We first checked by immunofluorescence whether β-Catenin was co-localized with autophagy-lysosomal organelles, using LC3 and LAMP1 as markers for autophagosomes and lysosomes, respectively. Butyrate increased the number of LC3-positive organelles and promoted their fusion with LAMP1-positive organelles (yellow signal), confirming the induction of autophagy and neo-genesis of autophagosomes (Figure 7A). This signal is particularly evident at one pole of the nucleus, which corresponds to the region where these two organelles meet at the micro-tubular organizing center. Double staining of β-Catenin/LC3 showed the co-localization of the two proteins, suggestive of the possible inclusion of β-Catenin within the autophagosomes (Figure 7A). To rule out any non-specific co-localization with the autophagosomal marker, we investigated whether β-Catenin co-localized with LAMP1. The images in Figure 7A show that β-Catenin indeed co-localized with LAMP1, which is suggestive of its translocation into lysosomes through autophagy. The quantitative analysis of the double-stained puncta is reported.

To definitively confirm that β-Catenin was sequestered (and eventually degraded) within the autophagy-lysosomal vacuoles, we performed a co-immunoprecipitation assay to check whether this protein indeed interacted with LC3 upon butyrate challenge. Data in Figure 7B show that butyrate promoted the binding between LC3 and β-Catenin, as indicated by the fact that β-Catenin was detectable only in precipitates containing LC3.

### 3.5. Disruption of Autophagy Prevents Butyrate-Induced β-Catenin Degradation and Inhibition of Cell Proliferation

**Figure 8:**
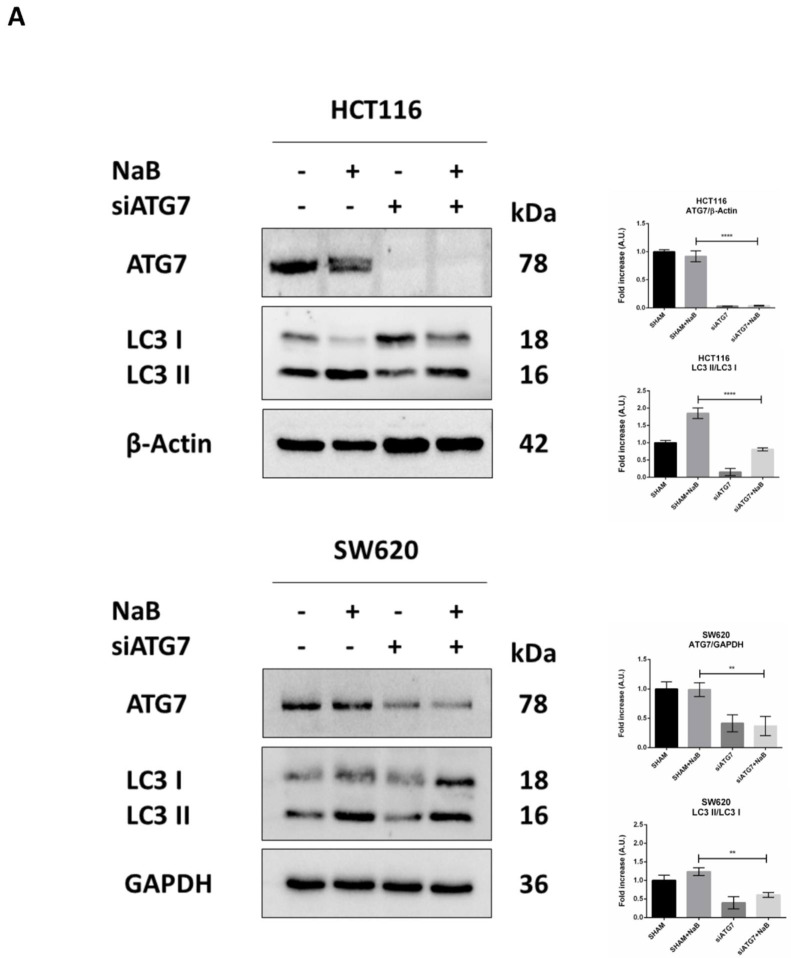

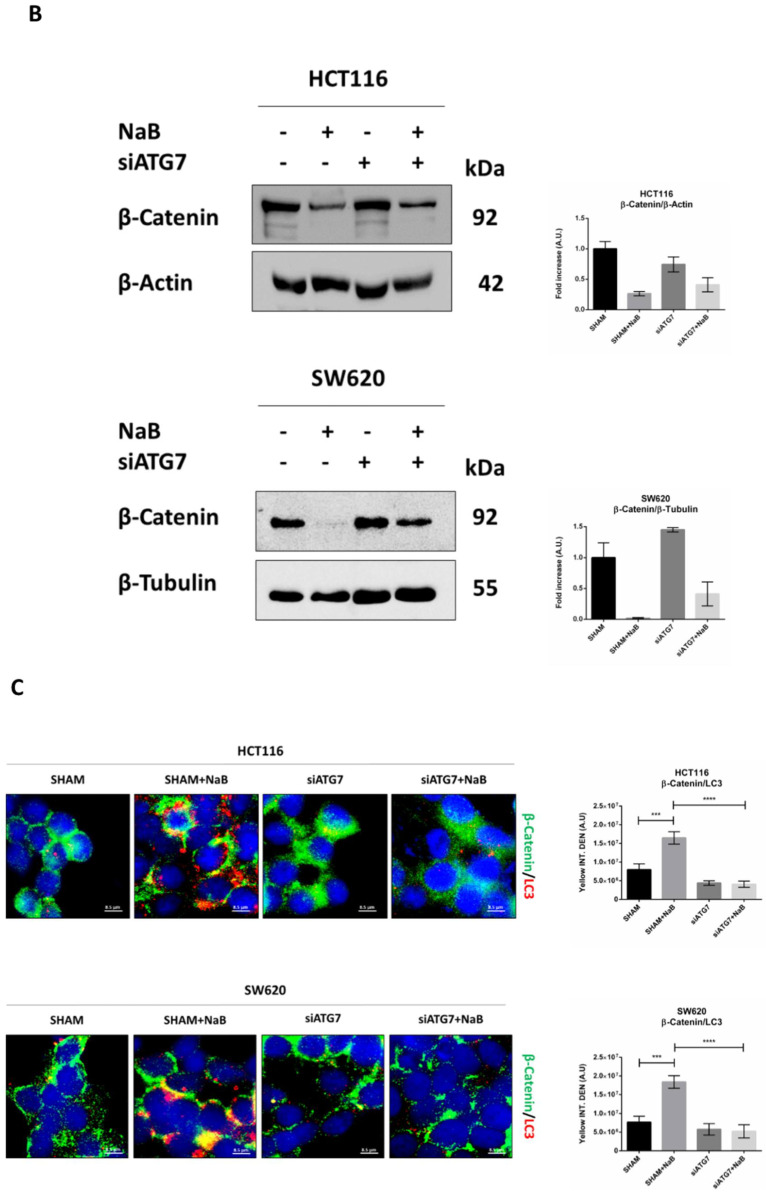

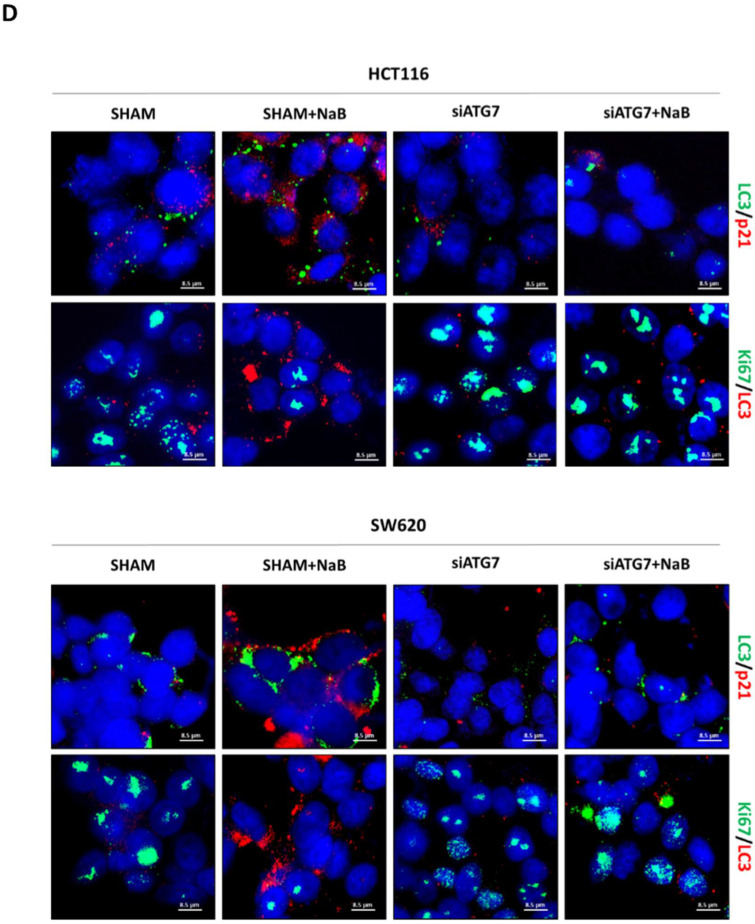
*ATG7* silencing prevents autophagy degradation of β-Catenin and restores CRC cell proliferation. HCT116 and SW620 cells adhering to petri dishes or on coverslips were transfected with siATG7 and treated or not with 2mM NaB. Medium was replaced, and substances re-added every 24 h. Western blotting (**A**,**B**) and immunofluorescence (**C**,**D**) analyses were performed at 48 h. (**A**) Expression of ATG7 and LC3; (**B**) expression of β-Catenin. (**C**) β-Catenin (green fluorescence)/LC3 (red fluorescence), (**D**) LC3 (green fluorescence)/p21 (red fluorescence) and Ki67 (green fluorescence)/LC3 (red fluorescence). Nuclei were stained with DAPI. Representative microscopic fields are shown (scale bar = 8.5 μm, magnification = 63×). Integrity density of yellow immunofluorescences, indicative of co-localization, is shown. The graphs represent the average ± SD and the yellow integrity density ± SD (significance was considered as follow: ** *p* < 0.01, *** *p* < 0.001, **** *p* < 0.0001).

Finally, we investigated whether autophagy degradation of β-Catenin functionally affected cell proliferation. To this end, we performed the genetic silencing of *ATG7*, which codes for one of the proteins involved in the post-translational lipidation of LC3-I mandatory for autophagosome formation. Effective inhibition of autophagy was indicated by the reduced level of ATG7 protein and accumulation of unprocessed LC3-I in siRNA-transfected cells (Figure 8A). When autophagy was disrupted by knocking down *ATG7*, the cellular level of β-Catenin was rescued in NaB-treated cells (Figure 8B).

In cells transfected with siATG7, the LC3 signal (red puncta) was greatly reduced, and this was paralleled by the decreased co-localization with β-Catenin (yellow puncta) and increased signal of the latter (green puncta) (Figure 8C). These effects were also observed in the presence of butyrate.

To verify whether the restoration of β-Catenin following autophagy disruption rescued cell proliferation, we assayed in the autophagy knocked-down cells the expression of Ki67, marker of cell proliferation, and of p21, marker of cell cycle block. To this end, we performed the immunofluorescences double-staining Ki67/LC3 and LC3/p21. Images show that the siATG7-transfected cells where formation of autophagosomes was efficiently prevented (as indicated by low LC3 puncta) also expressed a low level of p21 and increased level of Ki67, indicative of cycling cells (Figure 8D). These effects were also observed in cells exposed to butyrate.

Taken together, these data support the view that the knock-down of autophagy abrogates the butyrate-induced degradation of β-Catenin and results in the rescue of cell proliferation.

## 4. Discussion

The gut microbiota has been implicated in CRC development [36,37]. Microbiota metabolites such as short fatty acids (SCFAs) have shown beneficial effects in in vitro and in animal models of CRC [38]. Among SCFAs, butyrate is the one more abundant in the large intestine, where it acts as the major energy source for colonocytes and positively influences intestinal homeostasis [39].

Importantly, genetic alterations of the WNT/β–Catenin pathway play a pivotal role in the stepwise development of colorectal cancers [40]. In these cancers, cytoplasmic β-Catenin is undegraded, it accumulates, and is free to translocate into the nucleus, where it promotes the transcription of genes involved in cell cycle regulation [41]. Thus, it remains to be determined whether and how the microbiota metabolites can elicit a beneficial effect in such CRCs, whether this beneficial effect is linked to the disruption of the β-Catenin pathway and, if it is the case, which proteolytic pathway, alternatively to proteasome, is induced for β-Catenin degradation. Autophagy is an obvious candidate for the latter [42].

To address these issues, in the present study we employed two CRC cell lines defective in the proteasomal degradation of β-Catenin. In particular, the HCT116 cell line carries the S45del mutation of β-Catenin, resulting in the deletion of an amino acid at a GSK3β phosphorylation site, conferring a gain of function to β-Catenin [14,15], and the SW620 cell line carries the APC Q1338* mutation resulting in a premature truncation of the APC protein at amino acid 1338 and, thus, leading to a loss of APC protein function [16,17].

Given the critical role of the Wnt pathway in colorectal carcinogenesis [43], here we focused on β-Catenin function in cells bearing mutations affecting its turnover. A bioinformatic analysis of the genes and biochemical pathways correlated with *CTNNB1* (β-Catenin) expression performed on a database of CRC-affected patients showed that it is positively correlated to several genes involved in biological processes, which include mitotic cell cycle process, cell migration, epithelial-to-mesenchymal transition (EMT), regulation of gene expression; in contrast, *CTNNB1* expression negatively correlated with genes exerting a tumor suppressive role and genes involved in the autophagy-lysosomal pathway, particularly *ATG4D*. Starting from this correlation analysis and having proven that NaB can induce autophagy, we investigated the link between Wnt pathway and autophagy.

We tested the hypothesis that autophagy could mediate the beneficial effects of microbiota metabolites, using butyrate as representative of the latter. Here, we provide evidence that butyrate induces the growth arrest of cell proliferation in CRC cells bearing dysfunctional mutations of the *APC* and β-Catenin genes, and that this effect is mechanistically linked with the autophagy-mediated degradation of β-Catenin. We demonstrate that butyrate promoted the translocation of β-Catenin into autophagosomes and autolysosomes. In a co-immunoprecipitation assay, β-Catenin was not decreased with LC3 in control cells, indicating that, in this condition, it was free to translocate into the nucleus for the transcription of genes promoting cell proliferation. By contrast, in butyrate-treated cells, β-Catenin was precipitated along with LC3, indicative of autophagosome sequestration. Our finding agrees with the notion that β-Catenin contains an LC3 interacting domain (W/YXXI/L motif), which directs the target substrates for selective autophagy degradation [25]. Autophagy genetic knock-down resulted in the rescue of β-Catenin cytoplasmic level and a consequent restoration of the proliferation of CRC cells, proving that butyrate-induced autophagy degradation of β-Catenin was the mechanism for CRC growth arrest.

Of potential clinical relevance, butyrate could also elicit its beneficial effect in 3D spheroid cultures, which better resemble the in vivo growth of cancers, and even in the presence of IL-6, an inflammatory cytokine that is abundantly present in the tumor microenvironment and that has been reported to inhibit autophagy [44,45]. IL-6 is over-represented in the serum of CRC-affected patients [46], and its high level is associated with increased tumor growth and aggressiveness, and poor clinical outcome [47,48]. In CRC cells, IL-6 induced Cyclin D1 expression [34], and in 3D CRC spheroids stimulated the expansion of cancer stem cells [33]. Whether butyrate also induces the autophagy degradation of β-Catenin in the presence of this cytokine or whether it interferes with the IL-6 receptor remains to be determined. In this context, it is worth mentioning that autophagy impairment has been shown to lead to gut microbiota dysbiosis and to generate a chronic immune cell activation, which results in exacerbated intestinal inflammation, thus increasing the risk of CRC development and progression [20].

In summary, here, we demonstrated the functional role of autophagy in the protective and anticancer activities of the microbiota metabolite butyrate. This may open the way for novel approaches in the management of CRC. Currently, drug repositioning to inhibit both the Wnt/β-Catenin and autophagy pathways represents a new frontier of targeted cancer therapies [24].

Innovative therapeutic approaches, having reduced side effects and enhanced effectiveness for increasing the survival rate in colorectal cancer-affected patients, are urgently needed. Since diet affects the composition and the function of commensal bacteria, one adjuvant approach could be the use of dietary supplements that promote a “non-carcinogenic” microbiota and increase the probiotic strains producing butyrate.

## Figures and Tables

**Table 1:** Mutational status of relevant oncogenes and tumor suppressor genes. HCT116 cell line was isolated from the large intestine of colorectal cancer-affected adult male; SW620 cell line was derived from the lymph node metastatic site of colorectal cancer of a 51-year-old Caucasian male.

	*BRAF*	*KRAS*	*TP53*	*PTEN*	*APC*	*CTNNB1*
**HCT116**	I666I Silent P301P Silent	G13D (Missense)	Wild-type	Wild-type	Wild-type	S45del
**SW620**	Wild-type	G12V	R273H P309S	Wild-type	Q1338 (Nonsense)	NA

## Data Availability

The data that support the findings of this study are available from the corresponding author upon reasonable request.

## Supplementary Materials

The following supporting information can be downloaded at: https://www.mdpi.com/article/10.3390/biomedicines10051131/s1, Figure S1: Oncoprint of somatic mutation; Figure S2: SA-βgal activity.
