# Supplementary material for: Butyrate Inhibits Colorectal Cancer Cell Proliferation through Autophagy Degradation of β-Catenin Regardless of APC and β-Catenin Mutational Status
Source: Biomedicines. 2022 May 13;10(5):1131. doi: 10.3390/biomedicines10051131 (PMC9138675; doi:10.3390/biomedicines10051131)
Supplement: Supplementary file 1 [file biomedicines-10-01131-s001.zip › biomedicines-1680866-supplementary.pdf]

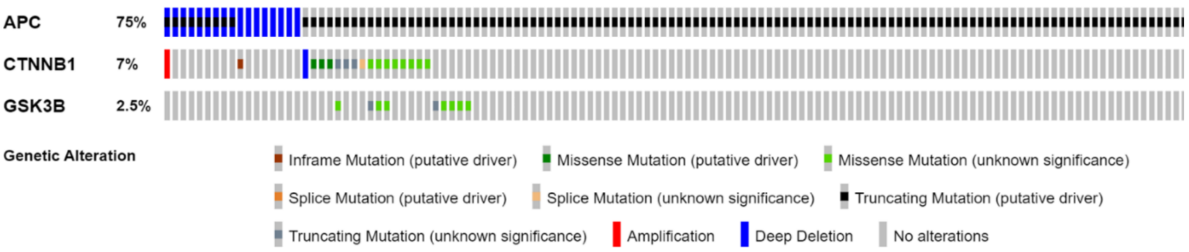

Figure S1. Oncoprint of somatic mutation. The oncoprint shows the genetic alterations and mRNA expression levels of *APC*, *CTNNB1*, and *GSK3β* in 526 colorectal adenocarcinoma (TCGA, PanCancer Atlas) patients.

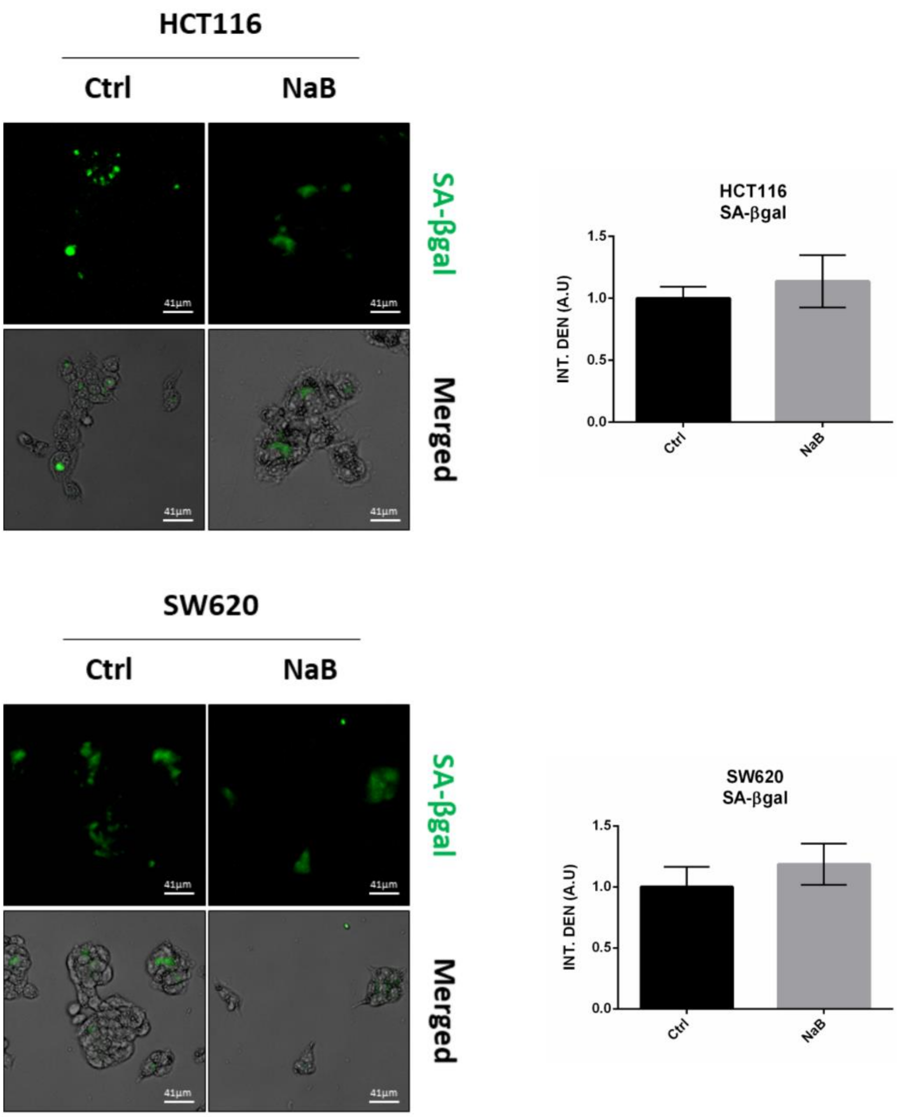

Figure S2. SA-βgal activity. HCT116 and SW620 cells were plated on 48-well plate and treated or not with 2mM NaB. Medium was replaced, and substances re-added every 24 h. Following 48 h, cells were incubated with β-galactosidase green fluorescent probe overnight. Representative images per each condition are shown. Fluorescence signal was quantified by using the ImageJ software and the graph represents the average fluorescence ± SD.
